# The Receptor CMRF35-Like Molecule-1 (CLM-1) Enhances the Production of LPS-Induced Pro-Inflammatory Mediators during Microglial Activation

**DOI:** 10.1371/journal.pone.0123928

**Published:** 2015-04-30

**Authors:** Aroa Ejarque-Ortiz, Carme Solà, Águeda Martínez-Barriocanal, Simó Schwartz, Margarita Martín, Hugo Peluffo, Joan Sayós

**Affiliations:** 1 Immunobiology Group, CIBBIM-Nanomedicine Program, Hospital Universitari Vall d’Hebrón, Institut de Recerca (VHIR), Universitat Autònoma de Barcelona, Barcelona, Spain; 2 Drug Delivery and Targeting Group, CIBBIM-Nanomedicine Program, Hospital Universitari Vall d’Hebrón, Institut de Recerca (VHIR), Universitat Autònoma de Barcelona, Barcelona, Spain; 3 Networking Research Center on Bioengineering, Biomaterials and Nanomedicine (CIBBER-BBN), Instituto de Salud Carlos III, Barcelona, Spain; 4 Department of Cerebral Ischemia and Neurodegeneration, Institut d’Investigacions Biomèdiques de Barcelona-Consejo Superior de Investigaciones Científicas (CSIC), Institut d’Investigacions Biomèdiques August Pi i Sunyer (IDIBAPS), Barcelona, Spain; 5 Biochemistry Unit, Faculty of Medicine, Institut d’Investigacions Biomèdiques August Pi i Sunyer (IDIBAPS), University of Barcelona, Barcelona, Spain; 6 Neurodegeneration Laboratory, Institut Pasteur de Montevideo, Montevideo, Uruguay; 7 Department of Histology and Embryology, Faculty of Medicine, UDELAR, Montevideo, Uruguay; Universidade de São Paulo, BRAZIL

## Abstract

CMRF35-like molecule-1 (CLM-1) belongs to a receptor family mainly expressed in myeloid cells that include activating and inhibitory receptors. CLM-1 contains two ITIMs and a single immunoreceptor tyrosine-based switch motif (ITSM), although also displays a binding site for p85α regulatory subunit of PI3K. By using murine primary microglial cultures, we show the presence of all CLM members in microglial cells and characterize the expression of CLM-1 both in basal conditions and during microglial activation. The TLR4 agonist lipopolysaccharide (LPS) and the TLR3 agonist polyinosinic–polycytidylic acid (Poly I:C) induce an increase in microglial CLM-1 mRNA levels *in vitro*, whereas the TLR2/6 heterodimer agonist peptidoglycan (PGN) produces a marked decrease. In this study we also describe a new soluble isoform of CLM-1 that is detected at mRNA and protein levels in basal conditions in primary microglial cultures. Interestingly, CLM-1 engagement enhances the transcription of the pro-inflammatory mediators TNFα, COX-2 and NOS-2 in microglial cells challenged with LPS. These results reveal that CLM-1 can acts as a co-activating receptor and suggest that this receptor could play a key role in the regulation of microglial activation.

## Introduction

Microglial cells, the only population of myeloid origin in the brain parenchyma, constitute the first line of defense of the central nervous system (CNS) [1]. In the healthy CNS, microglial cells are continuously surveying their local microenvironment [2]. Upon extracellular disturbances, microglia undergo a profound change of their functional phenotype from the resting/surveying state to an heterogeneous activation state, which has also been defined as effector state [3]. This effector state is characterized by an increase of the proliferation, migration, phagocytosis and the production of a broad range of both pro-inflammatory and anti-inflammatory mediators [4].

Although primarily beneficial, microglial activation has been postulated to have a detrimental effect when it becomes chronic and exacerbated, due to the release of potentially cytotoxic mediators such as reactive oxygen and nitrogen species, metalloproteinases (MMPs) and pro-inflammatory cytokines [5,6]. To prevent any potential damage to the nervous tissue, microglial activation is tightly regulated by the so-called “On” and “Off” signals [7]. Off signals are constitutively present in the brain parenchyma and are expressed mainly by healthy neurons, whereas On signals are expressed by endangered or impaired neurons [reviewed in 1,7]. On and Off signals include both soluble molecules, such as neurotransmitters or chemokines, and membrane bound molecules, and exert inhibitory or activating function by binding to receptors present in the membrane of microglial cells. The integration of all the inhibitory and activating inputs shapes the phenotype and response of microglial cells accordingly. In the last years, several members of the immunoglobulin superfamily such as CD200R, TREM-2 or SIGLECs have been reported to be key regulators of microglial activation, and its dysfunction has been implicated in different CNS pathologies such as Multiple Sclerosis or Alzheimer’s Disease [8–10].

CMRF-35–like molecules (CLM) constitute a family of type I transmembrane glycoproteins with a single extracellular IgG domain encoded by a cluster of genes located in mouse chromosome 11D [11]. This family comprises 9 members (CLM-1 to CLM-9), which are expressed mainly in cells of the myeloid lineage, with the exception of CLM-9 that is expressed by endothelial cells [12]. CLM-1 presents the a classical structure of a classic inhibitory receptor, with a long cytoplasmic tail that contains two immunoreceptor tyrosine-based inhibitory motifs (ITIMs) and a single immunoreceptor tyrosine-based switch motif (ITSM) [11]. Through the association of the phosphatase SHP-1 to its cytoplasmic tail, CLM-1 has been reported to inhibit osteoclastogenesis and to impair IgE-induced production of IL-6 by bone marrow derived mast cells (BMMCs) [11,13,14]. However, CLM-1 cytoplasmic tail also presents putative binding motifs for p85α, the regulatory subunit of PI3K and the adaptor protein Grb2 [14]. The presence of these motifs suggests a potential activating function of this receptor. In fact, CLM-1 has recently been shown to promote phagocytosis of dead cells through the binding to p85α [15]. Furthermore, CLM-1 has also been reported to deliver activating signals through the binding to the adaptor molecule FcRγ [14].

Regarding the role of CLM-1 in the CNS, a neuroprotective role for CLM-1 in a murine EAE (Experimental Autoimmune Encephalitis) model of multiple sclerosis was reported some years ago [16]. However, no detectable levels of CLM-1 receptor were found in CNS-resident microglial cells, being only present in invading monocyte/macrophages. On the other hand, we recently demonstrated the expression of CLM-1 and rCD300f, the rat ortholog of CLM-1, in primary cultures of microglia and oligodendrocytes and at lower levels in neurons [17]. In the present study, we further analyze the expression of CLM-1 in primary microglial cells and evaluate its role during microglial activation. Our findings demonstrate that microglial cells express two distinct isoforms of CLM-1, the full-length membrane-bound receptor and a soluble isoform. Furthermore, we report the role of the endogenous transmembrane isoform of CLM-1 as a co-activator receptor by its ability to enhance the production of LPS-induced pro-inflammatory mediators.

## Materials and Methods

### Antibodies and reagents

Monoclonal rat anti-CLM-1 primary antibody was from R&D systems (Abingdon, UK, catalog number MAB27881), armenian IgG hamster was from eBioscience (San Diego, CA, catalog number 16-4888-81), Alexa Fluor 594 chicken anti-rat secondary antibody was from Molecular Probes (Eugene, OR, catalog number A-21471) and goat anti-Armenian hamster IgG-FITC secondary antibody was from Santa Cruz Biotechnologies (Dallas, TX, catalog number sc-2446). Monoclonal hamster anti-CLM-1 antibody was a generous gift from Genentech (San Francisco, CA). E. Coli lipopolysaccharide (LPS) serotype 055:B5, Polyinosinic–polycytidylic acid (Poly I:C), Peptidoglycan (PGN), DAPI and Fluoromount were from Sigma (St.Louis, MO).

### Cell cultures

Animals were handled in accordance with the Guidelines of the European Union Council (86/609/EU), and the Spanish regulations for the procurement and care of experimental animals (1201 RD/2005, October 10) and the study was approved by the The Animal Care Committee of the Vall d'Hebron Institut de Recerca (VHIR), Barcelona, Spain (Associate permit number:5953).

Mixed glial cultures were prepared from 1- to 3-day-old neonatal C57BL/6 mice as described [18]. Briefly, cerebral cortices were dissected in phosphate buffered saline, carefully stripped of their meninges and digested with 0.25% trypsin in Hanks’ Balanced Salt Solution (Life technologies, Carlsbad, CA, USA) for 25 min at 37°C. Trypsinization was stopped by adding an equal volume of culture medium containing 0.02% deoxyribonuclease I (Sigma). The solution was pelleted (5 min, 200 g), resuspended in culture medium and brought to a single cell suspension by repeated pipetting followed by passage through a 105 m pore mesh. Cells were seeded at a density of 350.000 cells/mL (72.900 cells/cm^2^) and cultured at 37°C in humidified 5% CO_2_-95% air. Medium was replaced every 5–7 days. The culture medium used for all cultures consisted of Dulbecco’s modified Eagle medium-F-12 nutrient mixture (Life technologies, San Diego,CA), fetal bovine serum 10% (Lonza), penicillin 100 U/mL, streptomycin 100 g/mL (Life technologies) and amphotericin B 0.5 g/mL (Life technologies).

Mouse microglial cultures were prepared by mild trypsinization as described (Saura et al. 2003). Briefly, confluent mixed glial cultures were treated for 30 min with a low trypsin concentration (0.06%) in the presence of 0.25 mM EDTA and 0.5 mM Ca^2+^. This treatment results in the detachment of an intact layer of cells containing virtually all the astrocytes and leaves a population of firmly attached cells identified as >98% microglia. Twenty-four hours after isolation by this procedure microglial cultures were used.

For ELISA and crosslinking assays, isolated microglial cells were further trypsinized with 0.25% trypsin for 5 minutes at 37°C. Trypsinization was stopped by adding an equal volume of culture medium and cells were harvested. After centrifugation (5 min, 200 g), cells were resuspended in conditioned medium and seeded at a density of 400.000 cells/mL (83.300 cells/cm^2^). Twenty-four hours after isolation by this procedure microglial cultures were used.

COS-7 and NIH/3T3 (ATCC, catalog number CRL-1651 and CRL-1658 respectively) cells were grown in DMEM with 10% heat-inactivated FCS, 2 mM glutamine, 1 mM sodium pyruvate, 100 U/ml penicillin, and 100 μg/ml streptomycin. RAW264.7 cells (ATCC, catalog number TIB-71) were grown in RPMI 1640 medium supplemented with 10% heat-inactivated FCS, 2 mM glutamine, 1 mM sodium pyruvate, 100 U/ml penicillin and 100 μg/ml streptomycin.

### Cell treatments

For the experiments with the TLR agonists, microglial cells were treated with LPS (100 ng/mL), Polyinosinic:polycytidylic acid (poly I:C (10 μg/mL) and Peptidoglycan (PGN, 1 μg/mL) or vehicle. For functional assays anti-CLM-1 monoclonal antibody from hamster or IgG isotypic control (eBioscience) were added to the culture medium (5 μg/mL) and incubated at 37°C for 30 minutes. Cells were then treated with LPS (100 ng/mL), IL-4 (10 ng/mL, Preprotech) or vehicle.

### Cell transfection

COS-7 cells were seeded at 7 x 10^5^ cells in a 10 cm^2^ plate. After 24 hours, cells were transiently transfected using LyoVec (Invivogen) following manufacturer’s instructions.

### RNA isolation and amplification of CLM family members

RNA from cells was isolated using Purelink RNA micro kit following manufacturer’s instructions (Life technologies). RNA was retrotranscribed using High-Capacity cDNA Reverse Transcription Kit (Applied Biosystems) according to manufacturer’s instructions. For the detection of the transcripts of the CLM family members, oligos 3 to 18 were used (S1 Table) and PCR conditions were as follows: 94°C 3 min; 30 cycles of 94°C 1 min, 65°C 1 min, 72°C 1 min; and 72°C 10 min. DNA products were resolved in 1% agarose gels and visualized by ethidium bromide staining. Next, fragments were sequenced for confirmation under Big Dye cycling conditions on an Applied Biosystems 3730xl DNA Analyzer (Macrogen Inc).

### Semiquantitative PCR

For semiquantitative RT-PCR analysis of the full length and soluble isoforms of CLM-1, oligos 19–20 were used (S1 Table). 500 ng of RNA was retrotranscribed as explained above and PCR was performed using 50ng of cDNA as template under the conditions that follows: 94°C 3 min; 27 cycles of 94°C 1 min, 60°C 1 min, 72°C 1 min; and 72°C 10 min. DNA products were resolved in a 2% agarose gel and visualized by ethidium bromide staining. They were sequenced for confirmation as described before.

### Cloning of the full length and soluble isoforms of CLM-1

A PCR strategy was used to clone the full-length and soluble CLM-1 isoforms sequences from microglial mRNA. Oligos 1–2 in S1 Table, mapping to the 5- and 3- untranslated regions of CLM-1, were used for this purpose. PCR conditions were as follows: 94°C 3 min; 30 cycles of 94°C 1 min, 55°C 30 s, 72°C 2 min; and 72°C 10 min. DNA products were resolved in a 1% agarose gel and visualized by ethidium bromide staining. Expected size fragments were cloned into the pSpark cloning vector following kit instructions (Canvax, Cordoba, Spain) and sequenced for confirmation described before.

### Quantitative Real-Time PCR

Real-time PCR (QT-PCR) for CLM-1 transcript detection was performed using TaqMan gene expression assay for CLM-1. For the detection of CCL-17, COX-2, IL-1β, IL-6, TNFα, RELMα, NOS-2 transcripts, QT-PCR was carried out with the SYBR Green PCR Master Mix (Applied Biosystems). Primers were used at a final concentration of 200 nM (S1 Table). TaqMan probe of eukaryotic 18s was used for cycle normalization (Applied Biosystems). Samples were run for 40 cycles (30 secs 95°C, 1 min 60°C, 30 secs 72°C) on an ABI- Prism 7500 Sequence Detector (Applied Biosystems). Data were calculated by the 2-Delta or the 2 (Delta-Delta CT) Pfaffl methods, using as a normalizer 18s. All PCR reactions were set up in triplicates.

### Flow cytometry

Cell surface expression of the CLM-1 was tested by indirect immunofluorescence following standard techniques using a monoclonal anti-CLM-1 from hamster and the corresponding isotypic control [19]. Briefly, microglial cells were harvested and incubated with 5ug/mL of a monoclonal antibody from hamster or an isotypic control in PBS for 20 minutes at 4°C. After washing with PBS, cells were incubated with an anti-hamster-FITC in PBS for 20 minutes at 4°C (dilution 1:200). Samples were analyzed with BD FACSCalibur instrument and CellQuest Software (BD Biosciences).

### Immunofluorescence

Primary glial cultures were seeded on glass coverslips treated with poly-D-lysine (25 mg/L). Cells were fixed with 4% paraformaldehyde for 15 min at room temperature. Non-specific staining was blocked by incubating cells with 10% fetal bovine serum in PBS for 1 hour at room temperature. Monoclonal antibody rat anti-CLM-1 (R&D SYSTEMS, 20ug/mL) diluted in PBS containing 10% FBS was incubated overnight at 4°C. Next, coverslips were washed with PBS and were incubated with polyclonal chicken anti-rat alexa 594 (dilution 1:1000) and DAPI (for nuclei staining) in PBS with 10% FBS one hour at room temperature. Microscopy images were obtained with a Leica DM5000B microscope.

### ELISA

For the detection of soluble CLM-1, ELISA of supernatant from microglial cells treated with LPS (100 ng/mL) or vehicle was performed. Twenty four hours after treatment, supernatant was recovered. sCLM-1 (Cloud-Clone Corp, Houston, TX), PGE2 (Enzo Life Sciences, Farmingdale, NY), TNFα and IL-6 (Preprotech, Rocky Hill, NJ) protein levels were analyzed by ELISA following the instructions of the manufacturers.

### Data processing and statistical analysis

All results are expressed as mean + standard error of the mean (SEM). Student’s t-test was used to compare two experimental groups. One-way analysis of variance (ANOVA) followed by Newman-Keuls multiple comparison test was used to determine significant differences when three or more experimental groups with equal variances (F test) were compared. When significant differences in variance were detected the Kruskal-Wallis test was performed followed by Dunn's Multiple Comparison Test. Values of P <0.05 were considered statistically significant.

## Results

### Primary murine microglia expresses CLM1-8 mRNAs

**Fig 1:**
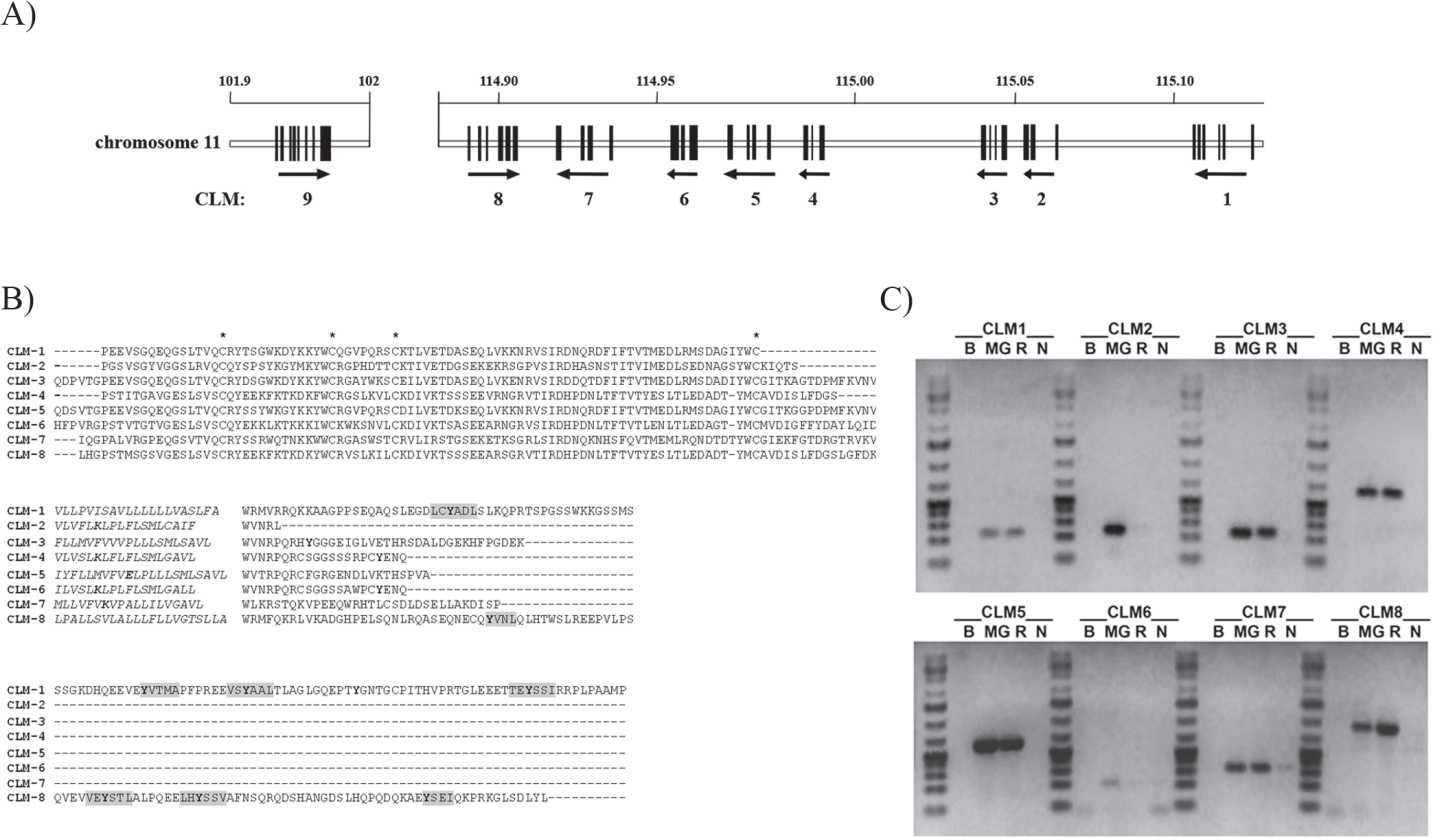
Microglia expresses all the members of CLM family. **A)** Genomic organization of CLM genes cluster at chromosomal region 11D. **B)** Sequence alignment of the Ig, transmembrane (cursive) and cytoplasmic domains of CLM members. Conserved cysteine residues are marked with asterisks. Transmembrane charged residues and cytoplasmic tyrosines are in bold type and ITIM-like sequences are in grey background. **C)** RT-PCR of CLM members was performed using specific primers on primary microglia total RNA (MG). Amplification from RAW264.7 (R) and NIH3T3 (N) cells lines was included as positive and negative controls, respectively. The image is representative 3 independent experiments.

The murine CMRF-35-like molecule (CLM) family is constituted by a cluster of eight genes on chromosome 11, encoding for activating and inhibitory cell membrane receptors expressed mostly by cells of the myeloid lineage (Fig 1A and 1B). With the aim to determine their expression pattern in murine primary microglia-purified cultures, mRNA was obtained and RT-PCR analysis was carried out using specific primers for each gene (S1 Table). As shown in Fig 1C, transcripts encoding for the eight CLM members were amplified from microglia. The mouse macrophage cell line RAW 264.7 was used as positive control, although CLM2 and CLM6 could not be detected. As expected, no amplification of transcripts occurred when using the fibroblast NIH3T3 cell line as template. PCR amplicons were sequenced to fully verify transcripts identity.

### CLM-1 receptor is expressed by murine primary microglia in basal conditions

**Fig 2:**
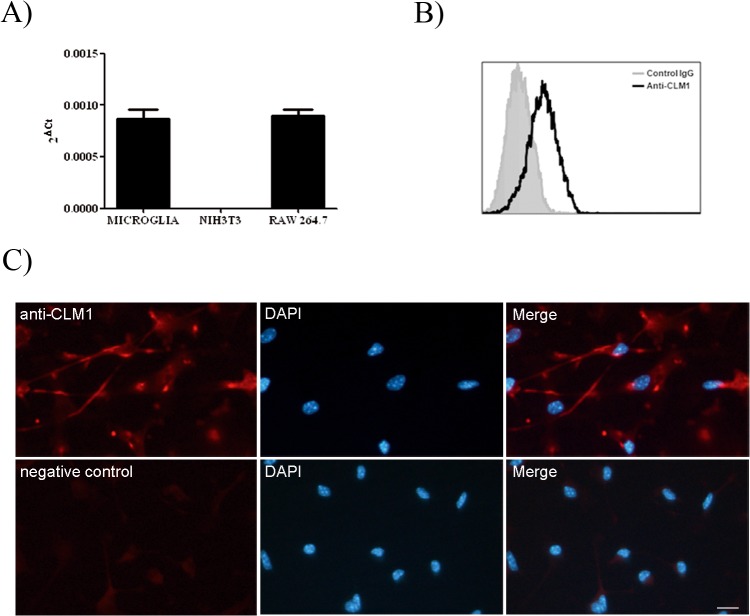
CLM-1 mRNA and protein levels in microglial cells under basal conditions. **A)** QT-PCR analysis of CLM-1 transcript was performed on primary microglia total RNA. RAW264.7 and NIH3T3 cell lines were used as positive and negative controls respectively. CLM-1 transcript was normalized with 18S RNA levels. **B)** Surface expression of CLM-1 on primary microglia was monitored by flow cytometry using anti-CLM-1 mAb (white histogram) and an isotypic mAb as a negative control (gray histrogram). **C)** Immunohistochemistry on microglial primary cultures was performed to analyze the expression of CLM-1 in basal conditions, using an anti-CLM-1 monoclonal antibody. Nuclei were stained with DAPI. No staining was observed in the absence of primary antibody. Scale bar, 20 μM.

To further characterize the expression of CLM-1 in microglia in basal conditions, RNA was extracted and CLM-1 mRNA levels were measured by quantitative RT-PCR (QT-PCR), which confirmed the presence of CLM-1 at similar levels as those found in the RAW264.7 cell line (Fig 2A). Next, protein expression of CLM-1 on the cell surface of microglial cells was assessed by flow cytometry analysis and immunofluorescence (Fig 2B and 2C). This antibody specifically recognizes CLM-1 and does not crossreact with any other CLM molecule expressed on the surface of transfected COS-7 cells (S1 Fig). Flow cytometry assay confirmed the presence of detectable levels of CLM-1 on the membrane of microglial cells in basal conditions (Fig 2B). As shown in Fig 2C, immunofluorescence staining in the absence of permeabilizing agents showed that the distribution of CLM-1 on the cell membrane is not homogenous, but organized in clusters *or* patches all along the cell surface. CLM-1 expression is affected during microglial activation.

**Fig 3:**
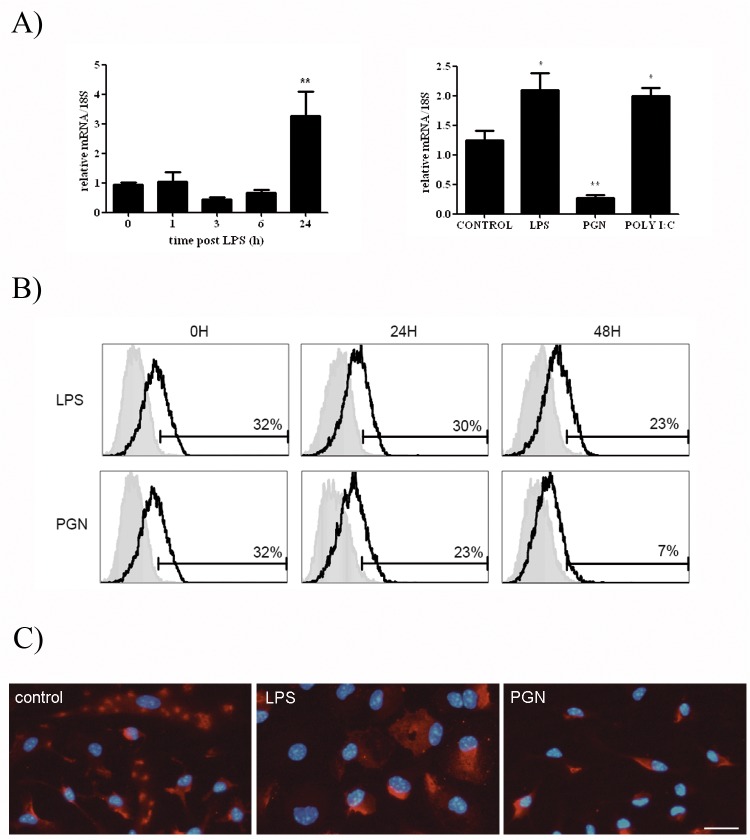
CLM-1 receptor expression levels are modified during microglial activation by TLRs agonists. **A)** Microglia was treated with LPS (100 ng/mL) at different time points (left) and with TLRs agonists LPS (TLR4 agonist, 100 ng/mL), PGN (TLR2/6 heterodimeragonist, 1 μg/mL) and poly I:C (TLR3 agonist, 10 μg/mL) for 24 hours (right). CLM-1 mRNA levels were quantified by QT-PCR. **B)** Surface expression of CLM-1 in microglial cells was monitored by flow cytometry 24 and 48 hours after treatment with LPS (100 ng/mL) or PGN (1μg/mL). Isotypic antibody (grey histograms), anti-CLM1 (white histograms). **C)** Immunostaining with an antibody for CLM-1 of microglia 24 hours after treatment with LPS (100 ng/mL) or PGN (1 μg/mL). Bar, 20 μM. Data are presented as mean ± SEM of 3 independent experiments. Statistically significant differences between treatments were determined by a one-way Anova followed by Newman Keules post-test. *P < 0.05 compared to IgG.

In order to determine whether CLM-1 expression levels were affected during microglial activation, primary microglia was treated with lipopolysacharide (LPS) at different time-points and CLM-1 mRNA levels were assessed by QT-PCR (Fig 3A). Whereas no differences were observed up to 6 hours after treatment, a significant increase in the amount of CLM-1 mRNA was detected 24 hours upon LPS treatment.

LPS is one of the most common inflammogens used to induce microglial activation, exerting its effects through the Toll-like receptor 4 (TLR4). To ascertain whether upregulation of CLM-1 could also be induced by other TLRs agonists, primary microglial cultures were treated with PGN, which is recognized by the TLR2/TLR6 heterodimer, and poly I:C, an analog of double-stranded RNA that binds to TLR3. RNA was extracted 24 hours after treatment and CLM-1 mRNA was measured by QT-PCR (Fig 3A). Similarly to LPS, poly I:C treatment produced an increase of CLM-1 mRNA expression. By contrast, PGN treatment induced a marked decrease in the amount of CLM-1 mRNA.

To determine whether the membrane protein levels of CLM-1 were also affected upon microglial activation with the TLR agonists, microglia was treated with LPS and PGN and flow cytometry analysis and immunofluorescence were performed. PGN-induced downregulation of CLM-1 was confirmed at protein levels by FACS staining, with a maximum effect 48 hours after treatment (Fig 3B). Unexpectedly, LPS treatment for either 24 hours or 48 hours did not result in the enhancement of CLM-1 expression on the membrane of microglial cells (Fig 3B). Immunostaining of microglial cells with an antibody against CLM-1 confirmed FACS data, since no increase in the intensity of CLM-1 immunoreactivity was seen after 24 hours of LPS treatment, whereas PGN induced a decrease of the fluorescence. However, it revealed a redistribution of CLM-1 in the microglial cells from the patched pattern in basal conditions to a highly polarized pattern in both LPS and PGN activated microglia (Fig 3C).

### Microglial cells express high levels of a soluble isoform of CLM-1

**Fig 4:**
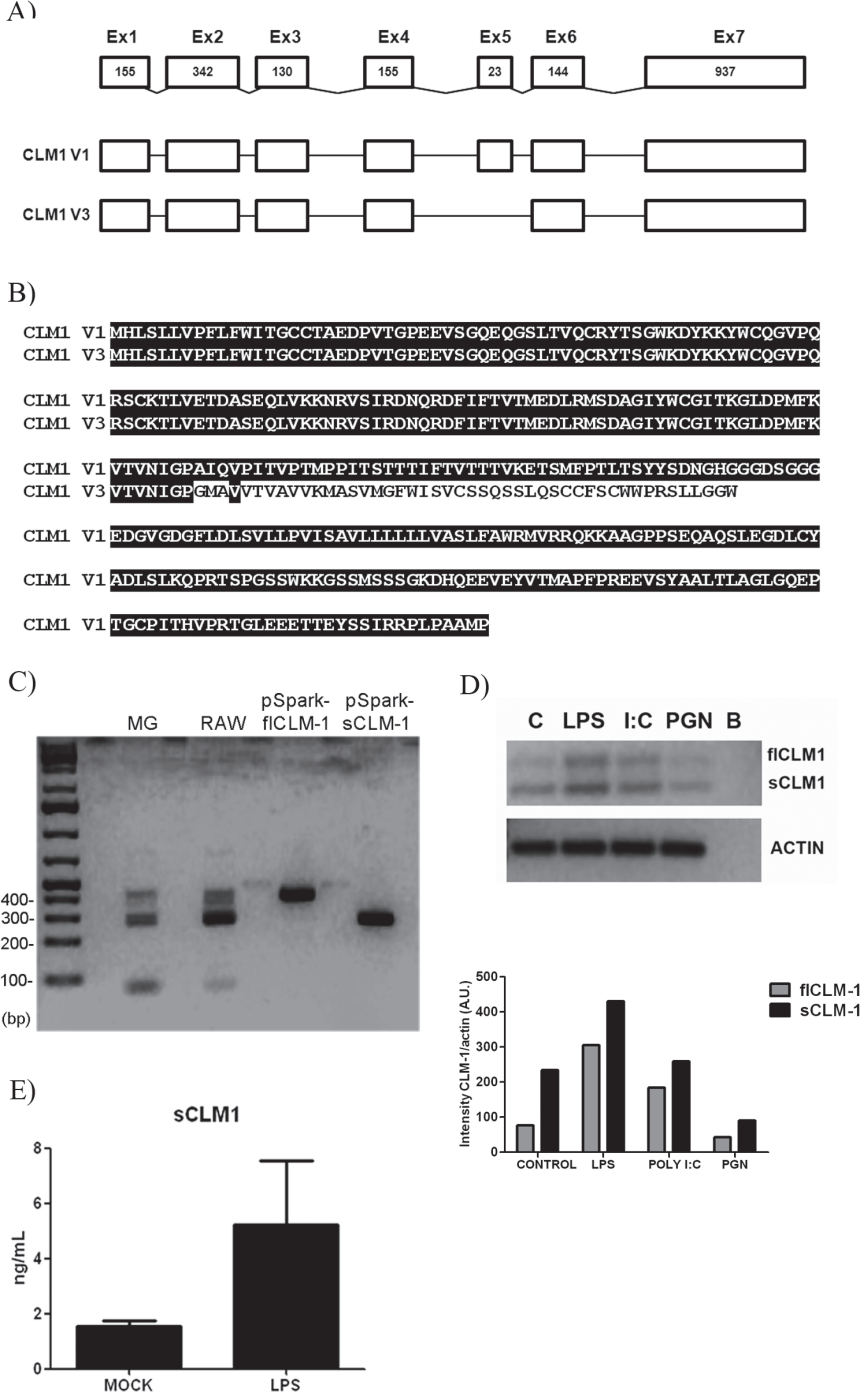
Microglia expresses a soluble isoform of CLM-1. **A)** Genomic organization and splicing pattern of CLM-1. A diagram representing CLM-1 splice variants 1, 2 and 3 is shown. Exons are represented by solid boxes (with their respective lengths in base pairs) and introns by the connecting lines. **B)** Comparison of the predicted amino acid sequences of CLM-1 variant 1 (NM_001169153) and variant 3 (JX073136). **C)** mRNA levels of the full length (variant 1) and soluble (variant 3) CLM-1 isoforms in microglia. Total RNA was extracted from primary microglia and RAW264.7 cells and RT-PCR was performed. As controls, cDNAs of both transcripts cloned in the vector pSPARK were used. The image is representative 3 independent experiments. **D)** Microglia was treated with the TLR agonists LPS (TLR4 agonist, 100 ng/mL), PGN (TLR2/6 heterodimer agonist, 1 μg/mL) and poly I:C (TLR3 agonist, 10 μg/mL) for 24 hours and RT-PCR analysis of the full length and the soluble isoforms of CLM-1 was performed. cDNAs were resolved in 2% agarose gels. The image is representative of 3 independent experiments. Actin mRNA levels were used as a loading control. Signal quantification is graphed bellow. **E)** Levels of soluble CLM-1 were measured in the supernatants of microglia after treatment with LPS (100 ng/mL) for 24 hours. Data are presented as mean ± SEM of 4 independent experiments. T-test was performed to determine significance and p-value was 0.1953.

Our laboratory has previously reported the existence of mRNA splicing variants for the human [20] and rat [17] CD300f gene, orthologs of CLM-1, encoding for putative soluble proteins lacking the transmembrane domain. With the aim to determine whether soluble isoforms were also produced from the murine CLM-1 gene in microglial cells, the CLM-1 genomic sequence was obtained from Ensembl mouse genome database (Ensembl BLAST Server at www.ensembl.org) and primers were designed (S1 Table) against the 5’ and 3’-UTR of the gene. Two cDNA fragments were cloned from microglial mRNA, one corresponding to the CLM-1 variant 1 or DigR2, which is translated into the full-length CLM-1 transmembrane receptor, and a new splicing variant (transcript variant 3) (Fig 4A). The new nucleotide sequence obtained (Gene Bank JX073136) was 1038 bp in length and contained an open reading frame of 526 bp that encoded for a 174 aa polypeptide that lacks the transmembrane region and consequently would be translated into a soluble protein (Fig 4B).

To ascertain whether the mRNA levels variations assessed by QT-PCR were due to the changes in the transcript of the soluble but not in the full length CLM-1, semiquantitative RT-PCR was performed from RNA from microglial cells treated with LPS, PGN and poly I:C for 24 hours. To this end, we designed a new pair of primers (S1 Table) flanking the nucleotide region that is not present in the soluble CLM-1 mRNA splice variant. The PCR products amplified from the full length and soluble CLM-1 mRNA (391bp and 261bp respectively) could be easily discriminated by electrophoresis. The two isoforms were detected in microglia as well as in RAW 264.7 cells and, surprisingly, the soluble variant was more abundant than the complete isoform in both cells types. Plasmids encoding cDNAs of the full length or soluble CLM-1 were used as positive controls (Fig 4C and S2 Fig). The identity of the PCR products was confirmed by sequencing. Semiquantitative RT-PCR revealed that PGN induced a marked decrease in the mRNA levels of both the full length and the soluble isoforms, whereas LPS and poly I:C induced an increase of CLM-1 mRNA levels, confirming the QT-PCR results (Fig 4D). Both the soluble and the full length mRNA levels were similarly affected upon treatment with LPS or poly I:C, ruling out the possibility that the increase in total mRNA observed without increase in cellular protein quantity would be due to increased transcription of the soluble mRNA variant (Fig 4D).

To further characterize the production of the soluble isoform of CLM-1 (sCLM-1) in microglia, culture media from microglial cells untreated or stimulated with LPS for 24 hours was recovered and levels of sCLM-1 protein were measured by ELISA. As shown in the Fig 4E, ELISA results confirmed that microglial cells do express and secrete the soluble isoform of CLM-1, but no statistically significant differences were found in the production of sCLM-1 by LPS-stimulated cells.

### CLM-1 receptor increases LPS-induced pro-inflammatory mediator production in primary microglial cells

**Fig 5:**
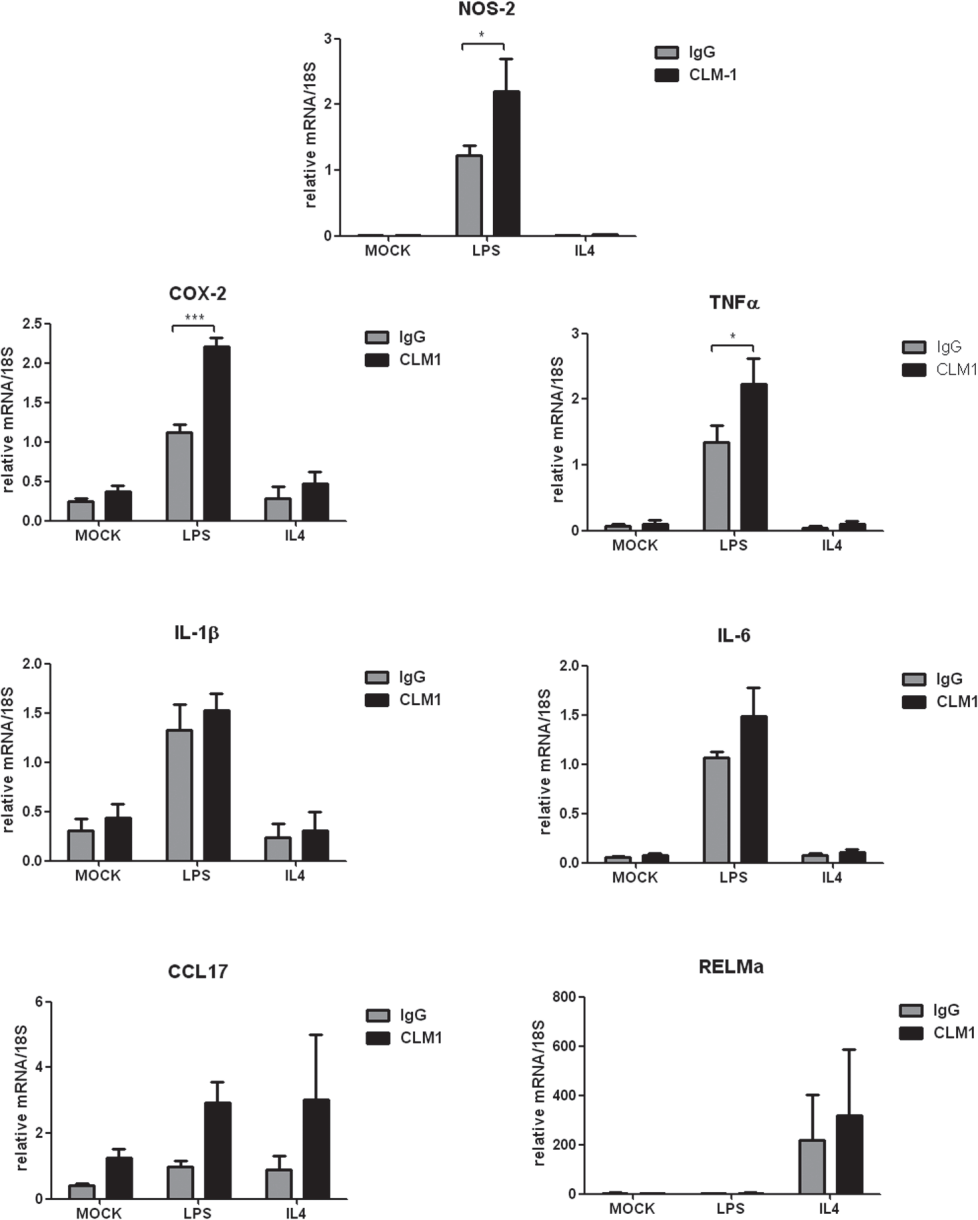
CLM-1 receptor enhances LPS-induced pro-inflammatory mediators production. Microglia was stimulated either with anti-CLM-1 Ab or an isotypic control in combination with LPS (100 ng/mL) or IL-4 (10 ng/mL). After 24 hours, total RNA was extracted and QT-PCR analysis was performed. Relative quantification was performed using the condition IgG+LPS as the calibrator condition for NOS-2, COX-2, TNFα, IL-1β, IL-6 and CCL17. For the quantification of RELMα, IgG+mock condition was used as the calibrator condition. Data are presented as mean ± SEM of 3 independent experiments. Statistically significant differences between treatments were determined by one-way ANOVA followed by Newman Keuls post-test, or Kruskal-Wallis test followed by Dunn's Multiple Comparison Test. *P < 0.05, **P<0.01 and ***P<0.001 compared to IgG.

Considering the potential dual inhibitory/activating function of CLM-1 reported in different cell types [13–15,21,22], we wondered which would be the outcome of triggering CLM-1 in microglial cells. To test this, CLM-1 receptor was engaged with a specific monoclonal antibody directed against CLM-1 or an isotype control antibody (5 μg/mL) in microglial cells differentiated to classical M1 (100 ng/mL LPS) or alternative M2 (20 ng/mL IL-4) phenotypes. Twenty-four hours after treatment, gene expression of the pro-inflammatory factors NOS-2, COX-2, IL-1b, IL-6 and TNFα as well as the M2-associated mediators Resistin-like molecule α (Relm-α) and CCL17 were measured by QT-PCR. Interestingly, the data showed that engagement of CLM-1 in microglial cells enhanced transcription levels of LPS-induced pro-inflammatory mediators NOS-2 and COX-2 (Fig 5). Furthermore, LPS-treated cells that were engaged with CLM-1 antibody also presented higher mRNA levels of the pro-inflammatory cytokines TNFα and IL-6 compared to those treated with the isotopic control antibody. In the case of the of CCL17 and Relm-α, no statistically significant differences were detected. No effects were observed at the mRNA level after triggering CLM1 activation in non-stimulated cells.

**Fig 6:**
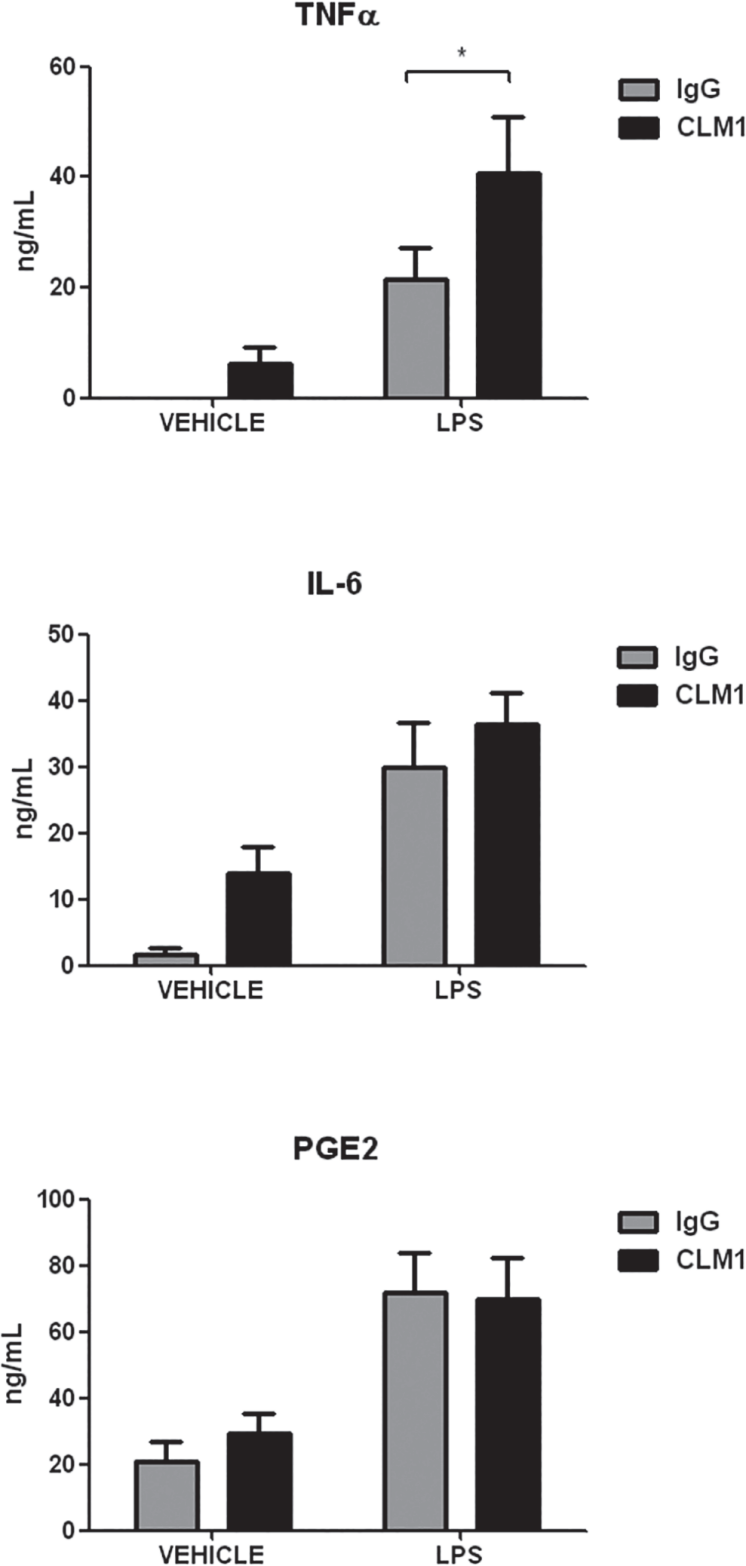
CLM-1 receptor amplifies LPS-induced TNFα protein levels. Microglia was stimulated either with anti-CLM-1 Ab or an isotypic control together with LPS (100 ng/mL). Supernatants were recovered 24 hours later and protein levels were measured by ELISA. Data are presented as mean ± SEM of 4 independent experiments. Statistically significant differences between treatments were determined by one-way ANOVA followed by Newman Keuls post-test. *P < 0.05 compared to IgG.

To further characterize the co-activating role of the CLM-1 receptor, protein levels of one of the main products of COX-2, PGE2, as well as the pro-inflammatory cytokines TNFα and IL-6 were quantified in the supernatant of LPS-treated microglial cells. LPS-induced TNFα production was enhanced in those cells where CLM-1 was engaged, whereas no differences were evidenced for PGE2 or IL-6 (Fig 6). Taking together, our data indicates that in our experimental conditions CLM-1 could act as a co-stimulator receptor in microglia.

## Discussion

In this report we have shown that cultured microglia express mRNA encoding for all the members of the CLM family, supporting the idea that these molecules could modulate the function of microglial cells. Focusing in CLM-1, we have demonstrated that this receptor is present on the surface of cultured microglia, and that its expression is modulated differently by diverse pro-inflammatory stimuli. The presence of CLM-1 mRNA and protein in microglia was reported in a previous work of our group [17], while another group reported that the receptor was not present in CNS-resident microglial cells [16]. A possible explanation for this discrepancy could be the different origin of the analyzed cells. Whereas in Xi et al. work microglial cells were isolated from spinal cord, our data has been generated using cells from brain cortex. Heterogeneity in the expression of some proteins among different brain regions has been reported by several groups [3]. For instance, microglial expression of TREM-2, a receptor highly homologous to CLM receptors, has been reported to vary both, inter- and intra-brain regions [23]. Another difference between both studies is that in one case CLM-1 expression was analyzed *in vivo* [16] whereas our data has been generated using microglial primary cultures. Microglia *ex vivo* displays a primed phenotype due to the lack of inhibitory signals that are present in the normal healthy CNS (reviewed [4,10]). Thus, we cannot discard the possibility of that CLM-1 expression is detected in our system due to an increase of CLM-1 levels when microglial cells are maintained under these conditionsOur results show that CLM-1 mRNA levels are upregulated when microglia is activated with the TRL4 agonist LPS. In our previous work [17], we also reported an increase of the mRNA levels of rCD300f, the rat ortholog of CLM-1, when microglial cells were challenged with LPS in combination with IL-1β [17]. CLM-1 increase after LPS treatment has also been reported in other cell types such as dendritic cells [24] and neutrophils [25]. Interestingly, the increase in CLM-1 mRNA levels does not seem a common feature of microglial activation, since not all the pro-inflammatory stimuli produce such increase. Treatment with LPS and the dsRNA analog poly I:C increase CLM-1 mRNA levels, whereas the TLR2/6 heterodimer agonist PGN induces a dramatic decrease of CLM-1 levels. The increase in CLM-1 mRNA occurs 24 hours upon LPS treatment, suggesting that it would act through an indirect mechanism, most probably by action of secreted factors which finally would affect CLM-1 in an autocrine manner. Engagement of TLR2/6 heterodimer by PGN triggers the Myd88-dependent signaling pathway whose major consequence is the transcription of pro-inflammatory cytokines, such as TNFα and IL-1β [26]. Additionally to Myd88-dependent pathway, TLR4 engagement by LPS induces the production of type I interferon cytokines IFNα and IFNβ through the activation of the TRIF-dependent pathway [26]. The TLR3 agonist Poly I:C also induces a type I IFN response by triggering the TRIF-dependent pathway [26]. More recently, Poly I:C has also been reported to bind to Mda-5, a RNA helicase enzyme that belongs to the RLR receptor family, which would finally induce type I IFN responses [27]. Thus, this difference in the cytokines secreted by microglia depending of the engaged receptor would finally be responsible of the increase or decrease of microglial CLM-1 mRNA levels reported in the present study. Although LPS-induced increase of CLM-1 transcript is consistent, we could only detect changes at protein levels in the cell membrane of microglia after PGN treatment, whereas no change could be detected even 48 hours after the treatment with LPS. Protein levels are known to be regulated both by transcription and translation mechanisms as well as post-translational degradative pathways. Thus, it is plausible that LPS treatment could affect some of those regulatory pathways ending with similar levels of CLM-1 protein in LPS-stimulated versus non-stimulated cells. Additional experimental data will be required to address this question.

One of the most interesting findings of our study is the description and detection of a new soluble isoform of CLM-1 (sCLM-1) in primary microglial cultures. Our laboratory reported the existence of two mRNAs encoding for putative soluble isoforms of CD300f, the human ortholog of CLM-1, in monocytes purified from PBMCs [20]. Furthermore, we recently have identified a presumed soluble isoform of rat CD300f receptor [17]. Interestingly, here we show for the first time that murine cells in culture produce a soluble isoform of CLM-1, even under basal conditions. Soluble isoforms of other members of the Ig superfamily such as TREM have been described. Soluble isoform of TREM-1 has been shown to attenuate the inflammatory response and improve survival in animal models of septic shock by inhibiting the TREM-1 receptor-mediated amplification of the inflammatory response in neutrophils and monocytes [28,29]. TREM-2 engagement increases the phagocytosis of neuronal debris and inhibits LPS-induced pro-inflammatory cytokines production responses in microglial cells [9,30]. The soluble isoform of TREM-2, has been reported to be increased in the cerebrospinal fluid of multiple sclerosis patients[31]and it is postulated that, as it happens with the sTREM-1, it would inhibit TREM-2 bound receptor function. Thus, we could postulate that sCLM-1, similarly to sTREM-1 or sTREM-2, might be counteracting the full-length CLM-1 receptor function acting as a decoy receptor. According with this, the use of CLM-1-Fc fusion protein and gene knock-out in EAE and Allergy models induced similar pro-inflammatory effects [13,16].

In the present work, we have studied for the first time the effect of endogenous CLM-1 during microglial activation. CLM-1 was first described by Chung et al. as an inhibitor of osteoclastogenesis [11]. It has also been reported to impair IgE-induced production of IL-6 by bone marrow derived mast cells (BMMCs) [13,14]. Interestingly, we demonstrate that engagement of microglial CLM-1 does not inhibit, but potentiates the production of the LPS-induced pro-inflammatory mediators COX-2, NOS-2, TNF-α and IL-6. These results are in accordance with recent studies that demonstrate that CLM-1 can also act as an activating receptor. Kitamura’s group demonstrated the dual functionality of CLM-1 in mast cells and how engagement of endogenous CLM-1 impairs cytokine production induced by FcεRI crosslinking or other TLRs agonists’ treatment, whereas it behaves as a co-activating receptor by enhancing LPS-induced IL-6 production [14]. CLM-1 receptor has also been recently reported to have activating potential promoting phagocytosis of apoptotic cells [15,32].

The mechanism by which CLM-1 potentiates LPS-induced pro-inflammatory phenotype of microglial cells remains unknown, although it could implicate the binding of the PI3K regulatory subunit p85α, which has been reported to mediate the CD300f-mediated stimulation of phagocytosis of apoptotic cells [15]. On the other hand, CLM-1 could also be triggering activating pathways in microglial cells through the binding to the adaptor protein FcRγ, which has been shown to bind to CLM-1 and to be responsible at least in part of the increase of LPS-induced IL-6 production in mast cells [14]. However, further studies would be needed to elucidate the exact mechanism responsible of the co-activating function of CLM-1 during microglial activation.

Taken together, these data demonstrate the expression in microglial cells in culture of the membrane bound receptor CLM-1 and a new soluble isoform, sCLM-1. Besides, we have described for the first time that CLM-1 as full-length receptor, acts as co-activator of TLR4 receptor by increasing gene expression of pro-inflammatory mediators in microglia upon LPS treatment. Our results, together with other reports reveal CLM-1 as a receptor functionally dual, key to finely tune microglial activation.

## Supporting Information

S1 Fig CLM-1 mAb from hamster specifically recognizes CLM-1. COS-7 cells were transfected with HA-tagged CLM-1 V1, CLM2, CLM4, CLM5, CLM6, CLM7, CLM8 or pDisplay empty vector. Forty-eight hours after transfection cells tested for CLM-1 Ab recognition by flow cytometry. Surface expression of the receptors was monitored using anti-HA (12CA5), anti-CLM1 and their corresponding isotypic antibodies as negative controls.
(TIF)

S2 Fig Agarose gel uncropped images of mRNA levels of the full length and soluble CLM-1 isoforms in microglia (left panel). Actin mRNA levels were used as a loading control (right panel).
(TIF)

S1 Table List of primers used in this study for RT-PCR and QT-PCR. (DOCX)
