# Supplementary figures and images for: The Receptor CMRF35-Like Molecule-1 (CLM-1) Enhances the Production of LPS-Induced Pro-Inflammatory Mediators during Microglial Activation
Source: PLoS One. 2015 Apr 30;10(4):e0123928. doi: 10.1371/journal.pone.0123928 (PMC4415817; doi:10.1371/journal.pone.0123928)

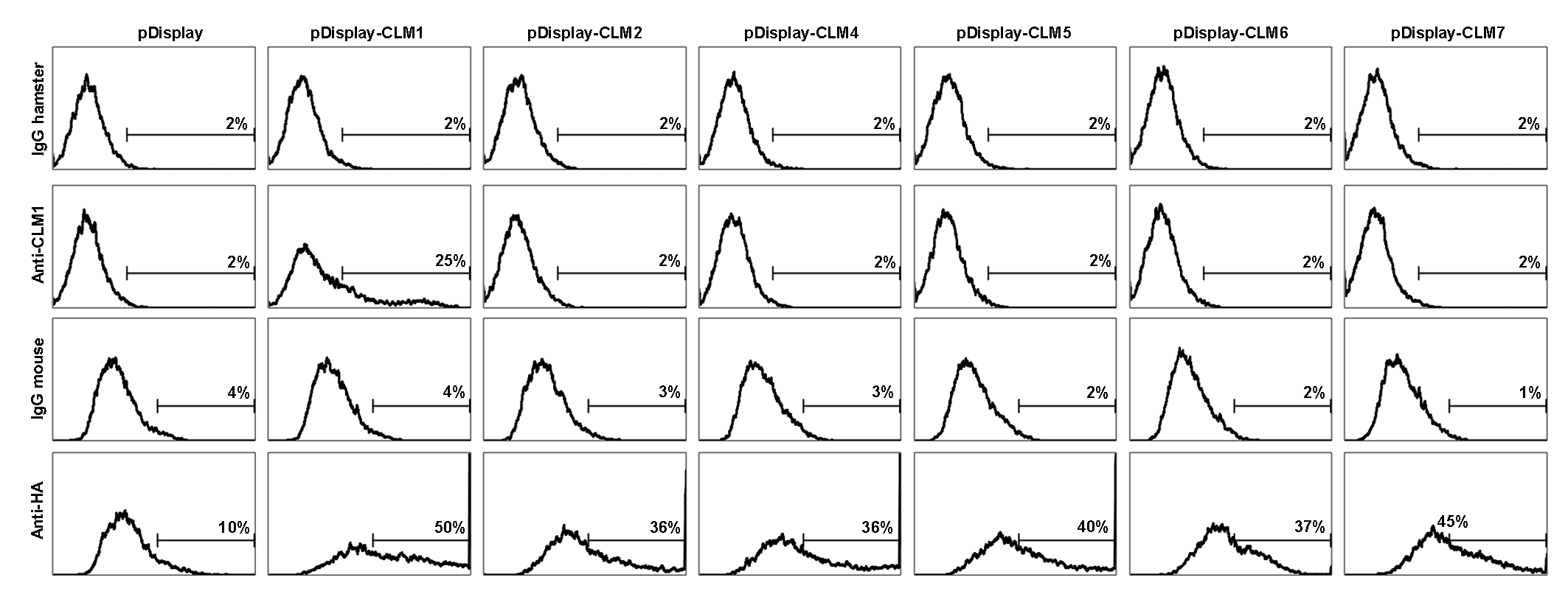

Supplement: S1 Fig — COS-7 cells were transfected with HA-tagged CLM-1 V1, CLM2, CLM4, CLM5, CLM6, CLM7, CLM8 or pDisplay empty vector. Forty-eight hours after transfection cells tested for CLM-1 Ab recognition by flow cytometry. Surface expression of the receptors was monitored using anti-HA (12CA5), anti-CLM1 and their corresponding isotypic antibodies as negative controls. (TIF) [file pone.0123928.s001.tif]

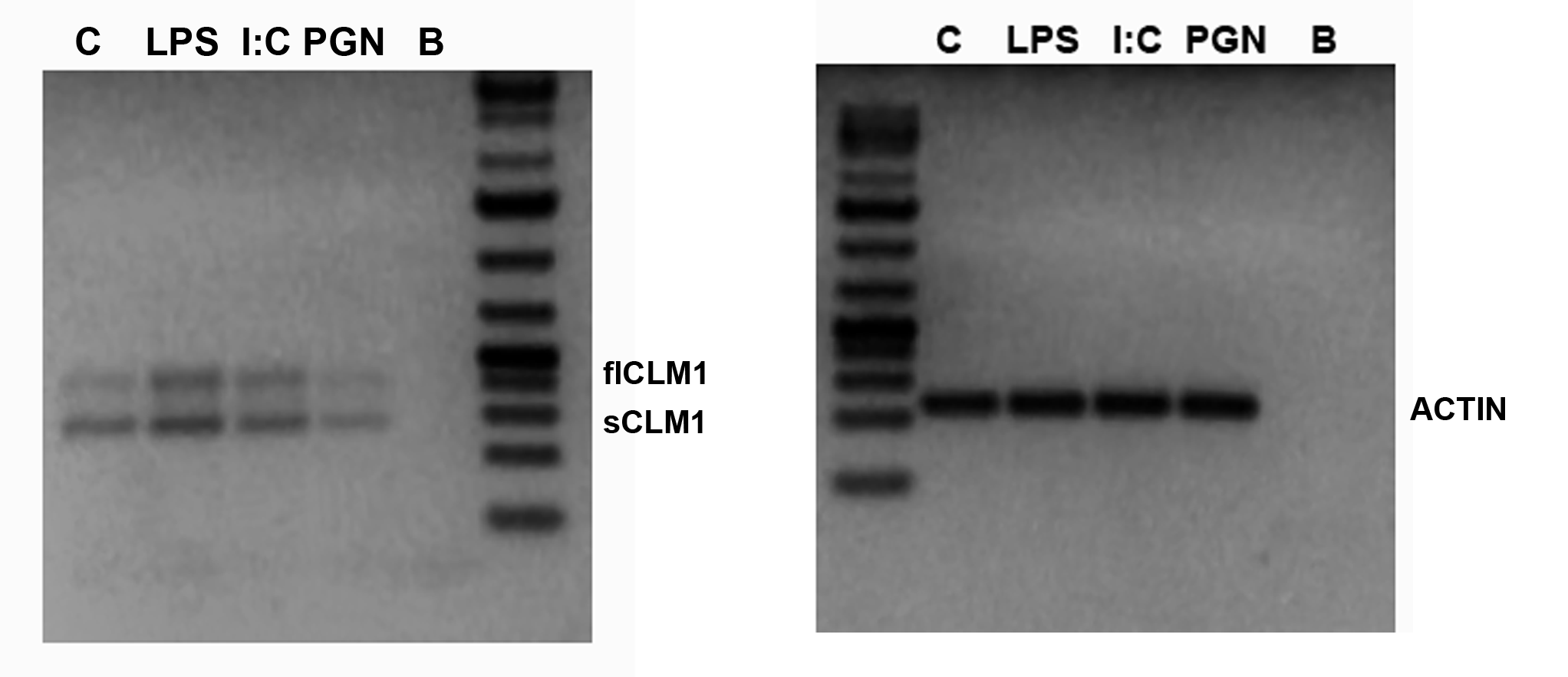

Supplement: S2 Fig — Actin mRNA levels were used as a loading control (right panel). (TIF) [file pone.0123928.s002.tif]
