# Supplementary material for: The Receptor CMRF35-Like Molecule-1 (CLM-1) Enhances the Production of LPS-Induced Pro-Inflammatory Mediators during Microglial Activation
Source: PLoS One. 2015 Apr 30;10(4):e0123928. doi: 10.1371/journal.pone.0123928 (PMC4415817; doi:10.1371/journal.pone.0123928)
Supplement: S1 Table — (DOCX) [file pone.0123928.s003.docx]

**Supplemental Table 1.**

|  | Molecule | Technique | Sense/  Antisense | Sequence 5’- 3’ |
| --- | --- | --- | --- | --- |
| 1 | CLM-1 | RT-PCR | Sense | GAAACCACAGGACCAGGAGA |
| 2 |  |  | Antisense | CGGGTAAGGAAGCAGGTACA |
| 3 | CLM-1 | RT-PCR | Sense | TCCAAGTACCCATTACAGTGCC |
| 4 |  |  | Antisense | CAGGACTGCAGAGATGACTGG |
| 5 | CLM-2 | RT-PCR | Sense | GTGCAGGTCTGCTCCTTCTC |
| 6 |  |  | Antisense | TCGAAGCATGGTCTCTGATG |
| 7 | CLM-3 | RT-PCR | Sense | GACCATGGAAGACCTGAGGA |
| 8 |  |  | Antisense | TGTTCTCTGTGCCAATGCTC |
| 9 | CLM-4 | RT-PCR | Sense | TGTGTCCCACTGCATGGC |
| 10 |  |  | Antisense | TCACTGGTTCTCATAACAG |
| 11 | CLM-5 | RT-PCR | Sense | CAGGATTCAGTCACAGGTC |
| 12 |  |  | Antisense | CTAGGCAACAGGACTATG |
| 13 | CLM-6 | RT-PCR | Sense | TGTCAAGACCAGTGCCTCAG |
| 14 |  |  | Antisense | GACCACGAACACCTCAACCT |
| 15 | CLM-7 | RT-PCR | Sense | TTACCATGGAGATGCTCAGG |
| 16 |  |  | Antisense | TCGCTACAGAGAGTGTGTCTCC |
| 17 | CLM-8 | RT-PCR | Sense | CTGCATGGTCCCAGCACC |
| 18 |  |  | Antisense | TCACAGGTAAAGGTCAGAG |
| 19 | flCLM-1 /sCLM-1 | RT-PCR | Sense | CAGGACTGCAGAGATGACTGG |
| 20 |  |  | Antisense | GCAGCTGGTGAAGAAGAACC |
| 21 | ACTIN | RT-PCR | Sense | CAACGAGCGGTTCCGATG |
| 22 |  |  | Antisense | GCCACAGGATTCCATACCCA |
| 23 | COX-2 | QT-PCR | Sense | CATCCTGAGTGGGGTGATGAG |
| 24 |  |  | Antisense | GGCAATGCGGTTCTGATACTG |
| 25 | CCL17 | QT-PCR | Sense | ACATAAAACGGCCTGTGA |
| 26 |  |  | Antisense | TTTGTGTTCGCCTGTAGTG |
| 27 | IL-1β | QT-PCR | Sense | TGGTGTGTGACGTTCCCATTA |
| 28 |  |  | Antisense | CAGCACGAGGCTTTTTTGTTG |
| 29 | IL-6 | QT-PCR | Sense | CCAGAGATACAAAGAAATGATGG |
| 30 |  |  | Antisense | ACTCCAGAAGACCAGAGGAAAT |
| 31 | NOS-2 | QT-PCR | Sense | GGCAGCCTGTGAGACCTTTG |
| 32 |  |  | Antisense | GGCAGCCTGTGAGACCTTTG |
| 33 | RELMα | QT-PCR | Sense | CCCTCCACTGTAACGAAGACTC |
| 34 |  |  | Antisense | CACACCCAGTAGCAGTCATCC |
| 35 | TNFα | QT-PCR | Sense | TGATCCGCGACGTGGAA |
| 36 |  |  | Sense | ACCGCCTGGAGTTCTGGAA |
